# Effect of Head Coverings on Directional Microphone Performance in Modern Hearing Aids: A KEMAR-Based Laboratory Evaluation

**DOI:** 10.3390/audiolres16050152

**Published:** 2026-10-03

**Authors:** Zahra Fekri, Mariam Mohamed, Ayasakanta Rout

**Affiliations:** Department of Communication Sciences and Disorders, James Madison University, Harrisonburg, VA 22807, USAroutax@jmu.edu (A.R.)

**Keywords:** directional microphone, directivity index, head covering, hijab, hearing aids, KEMAR, omnidirectional microphone, speech in noise

## Abstract

**Highlights:**

**What are the main findings?**

- No statistically detectable overall head covering effect on DI was observed across the four tested hearing aid systems.
- Covered–uncovered changes varied across frequencies and devices, but no frequency-specific contrast remained statistically significant after correction for multiple comparisons.

**What are the implications of the main findings?**

- The electroacoustic effect of the tested lightweight cotton covering was generally small on average but varied by frequency and hearing aid device; the study did not directly evaluate speech-in-noise benefit.
- Individual verification remains advisable because directional performance may vary across hearing aid devices, and the present findings are limited to the lightweight cotton configuration tested.

**Abstract:**

**Background/Objectives:** Directional microphones are an established hearing aid technology for improving speech understanding in background noise. Although researchers have examined the influence of hair, spectacles, and earmold venting on directional performance, no systematic work has addressed head coverings, which people wear worldwide for cultural, religious, occupational, and personal reasons. This study determined whether a cotton head covering alters the directivity index (DI) of highest-level receiver-in-canal (RIC) hearing aids. **Methods:** A Knowles Electronics Manikin for Acoustic Research (KEMAR) was positioned in a double-walled, sound-treated booth, and DI was measured for omnidirectional and fixed directional microphone settings, with and without a two-layer cotton covering placed over both microphone ports. We presented frequency-modulated tones at 500, 1000, 2000, and 4000 Hz at 60 dB SPL from a loudspeaker positioned 1 m from KEMAR, with all adaptive features disabled. We recorded root-mean-square levels at 30° increments and analyzed DI using a 2 × 2 × 4 repeated-measures analysis with microphone mode, head covering condition, and frequency as within-device factors. Covered–uncovered differences (ΔDI_H) were reported with 95% confidence intervals. **Results:** Fixed directional processing produced a mean horizontal-plane DI 1.50 dB greater than omnidirectional processing across the four tested hearing aid devices; however, this difference did not reach statistical significance when the hearing aid device was treated as the independent experimental unit, *F* (1,3) = 7.57, *p* = 0.071, partial η^2^ = 0.716. The overall covered–uncovered change was small (mean ΔDI = −0.023 dB, 95% CI: −0.193 to 0.148 dB; *p* = 0.699). The head covering × frequency interaction was not statistically significant after Greenhouse–Geisser correction (*p* = 0.301), nor was the head covering × microphone-mode interaction (*p* = 0.860). DI varied significantly across frequency (Greenhouse–Geisser adjusted *p* = 0.030). We did not perform inferential comparisons among hearing aid devices because only one system represented each manufacturer. **Conclusions:** No statistically detectable overall covering effect was observed across the four tested hearing aid devices; however, covered–uncovered differences varied by device and frequency. Given the small number of independent devices, the absence of statistical significance should not be interpreted as evidence of equivalence between covered and uncovered conditions. The findings are specific to the lightweight cotton configuration and laboratory conditions evaluated.

## 1. Introduction

Hearing is a complex sensory process that allows a listener to detect, locate, and identify sounds in a continuously changing environment. Under optimal conditions, the human auditory system can focus on a specific sound, such as a conversation partner’s voice, while ignoring competing noise. This ability, often called the “cocktail party effect”, depends on binaural processing, in which the two ears work together to distinguish pitch and timing and to differentiate the location of a particular sound [1]. In individuals with sensorineural hearing loss, these processes are disrupted: auditory filters broaden, temporal resolution declines, and speech becomes less distinct, making it much harder to follow conversations in noisy environments [2].

Hearing aids are the most common solution for reducing the communicative strain that hearing loss brings [3]. Modern digital hearing aids include several signal processing technologies, including noise reduction, feedback cancellation, frequency compression, and environmentally adaptive technology; nevertheless, studies over the past few decades continue to show that directional microphones remain one of the most established strategies for improving speech understanding in noise [4,5,6]. Directional microphones increase sensitivity to sounds arriving from the front of the listener while attenuating noise arriving from the sides and rear [6]. This selective sensitivity improves the signal-to-noise ratio (SNR) for the speech of interest, which typically originates from the conversation partner [5]. The spatial selectivity of a directional microphone can be characterized using a directivity index (DI), which compares sensitivity in a reference direction with sensitivity averaged across sound-arrival directions. Because the present measurements sampled only the horizontal plane, the analysis described below is reported specifically as a horizontal-plane directivity index [7]. Larger horizontal-plane DI values indicate greater spatial selectivity within the measured horizontal plane; they do not directly quantify behavioral speech-in-noise performance [8]. Both laboratory and field evaluations show that directional microphones yield better speech recognition than omnidirectional microphones, particularly when the conversation partner is in front of the listener and the competing noise is to the side or behind [4,9].

Directional microphone performance is dynamic and varies with several interconnected factors [10]. The angle and distance between microphone ports, the device housing configuration, the configuration of any vents, the angle of the user’s head, and the proximity of surrounding objects that temporarily block sound each influence the resulting polar pattern and therefore the DI [6,7]. Sound obstructed by hair, spectacles, or fabric may also be attenuated or scattered, with effects on performance that vary by frequency and depend on the weave density and thickness of the covering material. Although prior research has examined the effects of hair, spectacles, and earmold venting on directionality [7,10], no systematic study has explored the impact of head coverings on hearing aid DI. In everyday practice, some hearing aid users wear fabric head coverings for cultural, religious, medical, or personal reasons. These coverings vary considerably in material, thickness, number of layers, and placement relative to the hearing aid microphone ports. Any of these may deflect, attenuate, or scatter sound arriving at the microphone ports, particularly at higher frequencies, through absorption, diffraction, or scattering.

To date, the only study addressing this issue was conducted by Wagner [11], who evaluated the effect of hair covering on directional benefit in a small group of experienced hearing aid users. That study reported that a directional microphone provided a statistically significant benefit in noise compared with an omnidirectional microphone but found no statistically significant reduction in performance attributable to the hair covering. While these findings are clinically encouraging, the behavioral nature of the outcome measure, the small sample size, and the use of a single manufacturer limit the ability to isolate the acoustic mechanisms responsible for the observed robustness. Behavioral outcome measures may obscure subtle acoustic changes arising from individual differences among listeners, learning effects, or cognitive compensation, leaving it uncertain whether the preserved perceptual benefit corresponds to stable microphone polar patterns.

To evaluate whether head coverings could affect directional microphone performance, it is necessary to consider the acoustic mechanisms by which hair and fabrics interact with sound. These mechanisms include absorption, diffraction, and refraction, each of which can change how sound reaches the microphone ports.

Absorption refers to the loss of acoustic energy as sound travels through a material and is converted into heat. Hair exhibits frequency-dependent absorption and can act as a low-pass filter. Previous work indicates that higher frequencies are absorbed to a greater degree than lower frequencies and that absorption increases as hair becomes denser and heavier [11]. Citing prior acoustic research, Wagner [11] noted that thicker or denser hair samples show substantially greater absorption coefficients above approximately 2 kHz, whereas thinner or less dense hair produces little or no attenuation within the speech frequency range. Thin fabrics such as lightweight cotton likewise show very little absorption at low and mid frequencies but some attenuation of higher frequencies [12,13].

Diffraction is the bending of a sound wave around an obstacle. Low-frequency sounds have longer wavelengths and can therefore bend around the head and external coverings, whereas high-frequency sounds are more readily obstructed. Because directional microphones derive much of their selectivity from mid- to high-frequency cues, substantial diffraction or shadowing at these frequencies could reduce directional benefit. For fabric coverings, the magnitude of acoustic effects is more likely to depend on material thickness, fiber structure, porosity or air permeability, number of layers, and the fabric’s proximity to the hearing aid microphone ports.

Refraction is a change in the speed or direction of sound as it passes from one medium to another with different acoustic impedances. In principle, this can disrupt the timing relationships between microphone ports that are essential for directional processing. Wagner [11] hypothesized that hair covering could interfere with directional processing through refraction, but the behavioral results indicated that any such disruption was insufficient to produce detectable degradation of directional benefit.

Physically, thick or multilayered fabrics may block higher frequencies and obscure spatial information, thereby reducing DI [5,6]. In contrast, lightweight, open-knit or perforated materials may have a negligible effect. The absence of objective data prevents audiologists from giving specific recommendations to patients who regularly wear cultural or protective head coverings, and limits knowledge of whether a particular directional configuration will maintain the intended benefit in the wearer’s typical listening environment.

Given the widespread use of such coverings and the important role of directional microphones in hearing-in-noise benefit [4], this study aimed to objectively assess the effect of a two-layer cotton covering on the horizontal-plane DI of four highest technology level RIC hearing aid devices. Using a KEMAR in a controlled laboratory setting isolates microphone performance from listener-related variables and provides a mechanistic evaluation of how head coverings affect the spatial selectivity underlying directional benefit.

## 2. Materials and Methods

### 2.1. Study Design and Rationale

A 2 × 2 × 4 repeated-measures laboratory design was used to evaluate directivity index as a function of microphone mode (omnidirectional vs. fixed directional), head covering condition (uncovered vs. covered), and test frequency (500, 1000, 2000, and 4000 Hz). A KEMAR (GRAS Sound & Vibration A/S, Holte, Denmark) was used to eliminate human variation (for example, hearing loss, attention, and head movement) and to isolate microphone performance. No human participants were involved.

### 2.2. Test Environment

**Figure 1:**
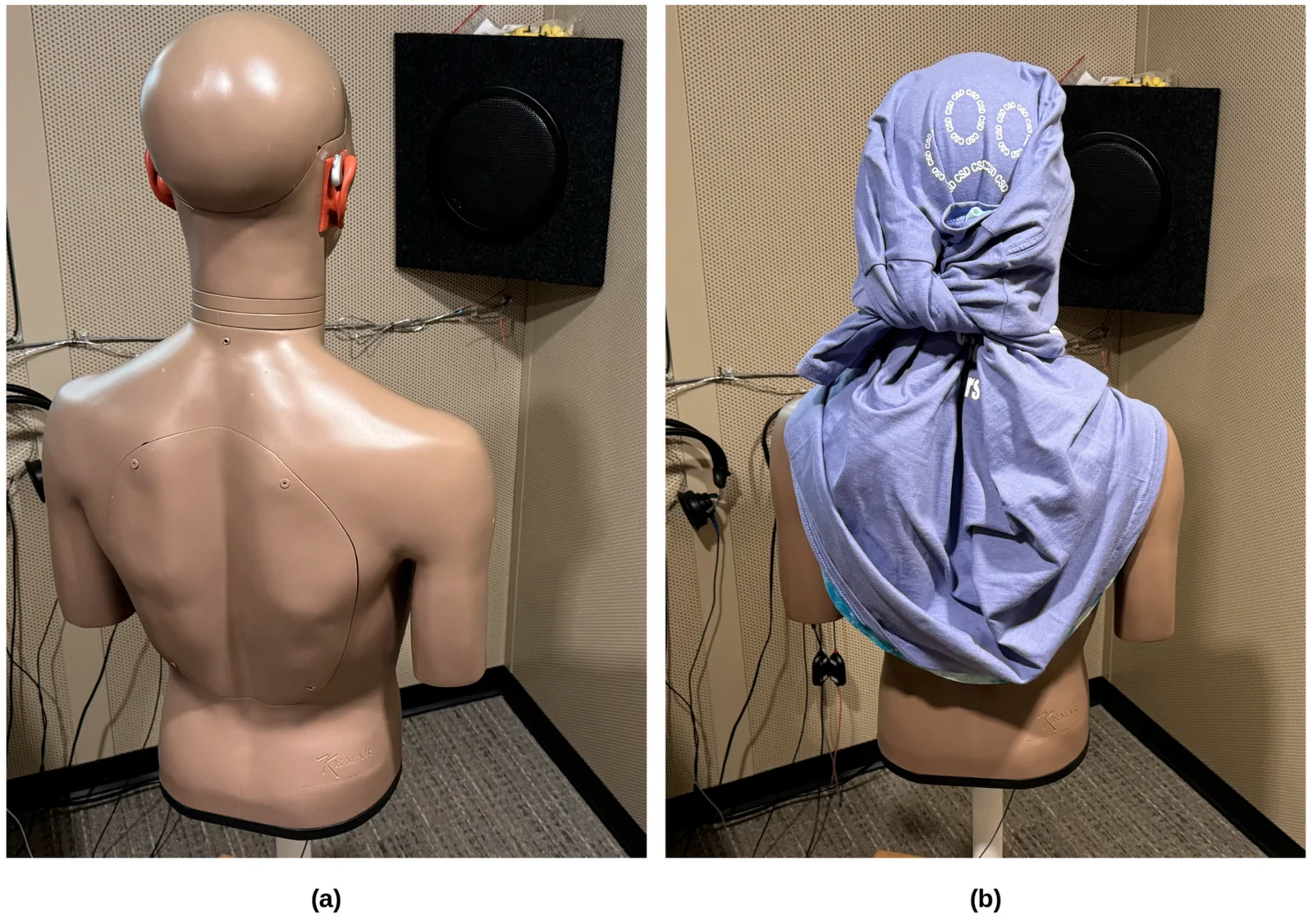
Experimental measurement configuration in the double-walled sound-treated booth, viewed from behind KEMAR with the loudspeaker positioned 1.0 m from the center of KEMAR’s head. (**a**) Uncovered condition, showing the receiver-in-canal hearing aid device fitted on KEMAR’s right ear. (**b**) Covered condition, with the lightweight cotton covering wrapped over the head and both ears so that the hearing aid microphone ports were fully covered.

Testing was conducted in a double-walled, sound-treated booth (IAC Acoustics, North Aurora, IL, USA; 2.40 m × 3.00 m). The booth was not an anechoic chamber and therefore cannot be assumed to satisfy free-field requirements for standardized directivity measurements. Reflected energy within the booth may have influenced the measured polar patterns and the depth of directional nulls. Accordingly, the present measurements are interpreted as comparative electroacoustic responses obtained under a consistent laboratory configuration rather than as standards-conforming anechoic measurements. Stimuli were presented through a loudspeaker positioned 1 m from KEMAR. The loudspeaker remained fixed at 1.0 m from the center of KEMAR’s head throughout testing. The frontal reference region was defined by the 30° and 60° measurement positions in the angular coordinate system used during data collection; responses at these two adjacent azimuths were averaged to calculate the horizontal-plane directivity index. KEMAR was rotated clockwise in 30° increments through 360°, while the loudspeaker position and source-to-head distance remained unchanged. The original experimental protocol did not include independent measurements of room reverberation time or reference-system directivity; therefore, reflected energy could not be quantitatively excluded from the measurements. The test set-up, with and without the head covering in place, is shown in Figure 1.

### 2.3. Hearing Aids and Programming

Four pairs of premium-level RIC hearing aids, defined as the highest-technology models offered by their respective manufacturers at the time of testing, were evaluated and designated Device 1, Device 2, Device 3, and Device 4. Manufacturer and model identifiers are not reported because the devices were evaluated under an agreement requiring manufacturer anonymity. Device numbers serve only as labels and do not imply any ranking of performance. This study examined the effects of the head covering and microphone directionality within each device, rather than comparing performance across manufacturers or devices. All devices were programmed for a bilateral, symmetrical sloping loss (45 dB HL at 250 Hz to 70 dB HL at 8000 Hz) using NAL-NL2 targets in each device’s fitting software via Noah 4 (HIMSA, Copenhagen, Denmark). Two programs were created for each device: an omnidirectional program and a fixed directional program. Directional mode was selected manually within each device’s fitting software, and automatic microphone switching was disabled. All adaptive features that could alter the microphone response, including noise reduction, feedback suppression, and automatic microphone switching, were disabled to ensure stable, repeatable polar responses. Compression and other signal processing parameters were set by each manufacturer’s fitting software for the NAL-NL2 prescription. All devices were behind-the-ear units in a receiver-in-canal configuration, which provides microphone port spacing typical of commercially available units (approximately 4–10 mm, although spacing greater than 13 mm is optimal for directionality) [14]. Both hearing aids in each pair were programmed and fitted bilaterally on KEMAR; however, acoustic recordings were obtained only from the right KEMAR ear. Binaural wireless communication between the two hearing aids remained at the manufacturers’ default settings so that each pair could operate as a linked bilateral fitting; consequently, any contribution of binaural coordination to the right-ear response is included in the measured values and was not isolated as a separate factor.

### 2.4. Head Covering Material

**Figure 2:**
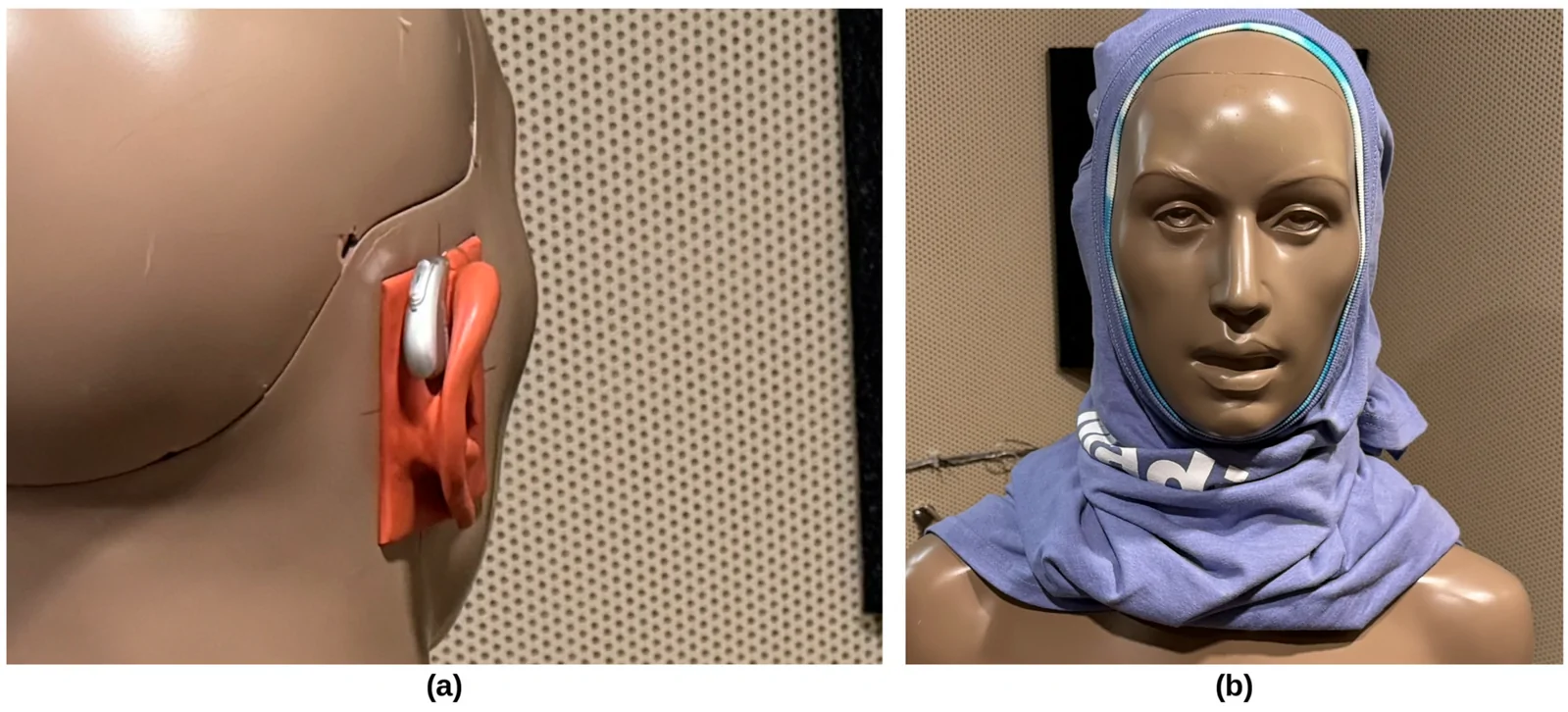
(**a**) Close-up of the hearing aid device positioned on KEMAR’s right ear, the ear from which recordings were obtained, before the covering was applied. (**b**) Frontal view of the lightweight cotton covering in place, showing symmetrical coverage of both ears and the hearing aid microphone ports.

The head covering condition was simulated using two layers of cotton fabric, each with an areal mass of approximately 150–180 g/m^2^ (4.4–5.3 oz/yd^2^). The fabric was used as a standardized experimental covering rather than as a model of any specific garment or head covering morphology. The material completely covered the hearing aid microphone ports during the covered condition [12]. The fabric was wrapped over the hearing aid microphone ports firmly enough to provide secure coverage, but not so tightly as to cause audible feedback. Cotton was selected as a lightweight experimental fabric rather than as a material intended to represent all cultural or personal head coverings. The study therefore evaluated the effect of this specific lightweight cotton configuration on hearing aid directional response. Previous acoustic studies indicate that fabric sound transmission and absorption depend on material properties, including thickness, density, porosity, and number of layers [15,16]. Both fabric layers were positioned uniformly around KEMAR so that fabric placement did not vary between the two sides of the head during testing. Both microphone ports were completely covered by the fabric (Figure 2).

### 2.5. Stimuli and Recording Procedure

Stimuli were frequency-modulated tones at octave frequencies from 500 to 4000 Hz presented at 60 dB SPL. The frequency-modulated tones were generated using a GSI audiometer (Grason-Stadler, Eden Prairie, MN, USA) and, according to the manufacturer’s specifications, had a modulation rate of 5 Hz, a frequency deviation of ±5% around each center frequency, and rise/fall times of approximately 20–50 ms. Presentation level was verified using a TSI Quest SoundPro (TSI Incorporated, Shoreview, MN, USA) Type 1 sound level meter (a precision grade instrument meeting the Type 1/Class 1 accuracy requirements of ANSI S1.4 [17] and IEC 61672-1 [18]), calibrated before testing with a Quest QC-10 acoustic calibrator (TSI Incorporated, Shoreview, MN, USA). The hearing aids were powered on throughout data acquisition and remained in the programmed test condition during each measurement sequence. KEMAR was rotated in 30° increments through 360°, and hearing aid output was recorded using an Etymotic ER-11 system (Etymotic Research, Elk Grove Village, IL, USA) and Sony Sound Forge 9.0 (Sony Creative Software, Madison, WI, USA). At each azimuth, hearing aid output was recorded for 30 s. A 6-s steady-state segment of each recording was selected for RMS analysis. Each experimental condition was recorded twice as a technical replicate. The hearing aids and fabric remained in the same position between the two recordings. Therefore, the repetitions quantify acquisition repeatability rather than variability associated with repositioning.

### 2.6. Directivity Index Calculation

Root-mean-square (RMS) output levels at each measured azimuth were converted from decibels to linear mean-square values before calculation of the directivity factor. For each frequency, hearing aid device, microphone mode, head covering condition, and technical repetition, the frontal reference response was defined as the average of the responses measured at 30° and 60°. These two adjacent azimuths represented the frontal reference region in the angular coordinate system used during data collection. The horizontal-plane directivity factor, Q_H_, was calculated as the ratio of this frontal reference response to the spatially averaged response across all measured horizontal-plane azimuths. The horizontal-plane directivity index was then calculated as:

DI_H_ = 10 log_10_ [Q_H_]

DI_H_ was calculated separately for each technical repetition; the two repetitions were retained to assess repeatability and averaged within each experimental condition for inferential analysis. Because measurements were restricted to the horizontal plane and were obtained in a sound-treated rather than an anechoic environment, the resulting values are interpreted as horizontal-plane directivity indices specific to the present measurement configuration rather than as full standards conforming spherical directivity indices [19].

### 2.7. Statistical Analysis

Each of the four hearing aid devices was treated as one independent experimental unit. The two recordings obtained for each measurement condition were considered technical repetitions and were not treated as independent hearing aid devices. Directivity index was calculated separately for each repetition, and the two technical repetitions were subsequently averaged within each device, frequency, microphone mode, and head covering condition for inferential analysis.

A repeated-measures analysis was conducted with microphone mode (Omni vs. Dir), head covering condition (uncovered vs. covered), and test frequency (500, 1000, 2000, and 4000 Hz) as within-device factors. Greenhouse–Geisser corrections were applied to effects involving the four-level frequency factor when appropriate.

The effect of the head covering was additionally quantified directly as:

ΔDI = DI covered − DI uncovered

for each device, frequency, and microphone mode. The absolute magnitude of the change, |ΔDI|, was also examined to characterize covering-related changes regardless of direction. Planned frequency-specific covered–uncovered contrasts were performed separately for Omni and Dir modes, with 95% confidence intervals and Cohen’s *d*_z_ reported. Holm adjustment was applied to account for multiple frequency-specific comparisons.

Because only one hearing aid device represented each manufacturer, differences among the four tested devices were described descriptively, and no device-level inferential comparisons were performed. Given the small number of independent devices (*n* = 4), emphasis was placed on effect estimates and 95% confidence intervals in addition to *p*-values. Statistical significance was set at α = 0.05. Analyses were conducted using IBM SPSS Statistics version 30 (IBM Corp., Armonk, NY, USA).

## 3. Results

**Figure 3:**
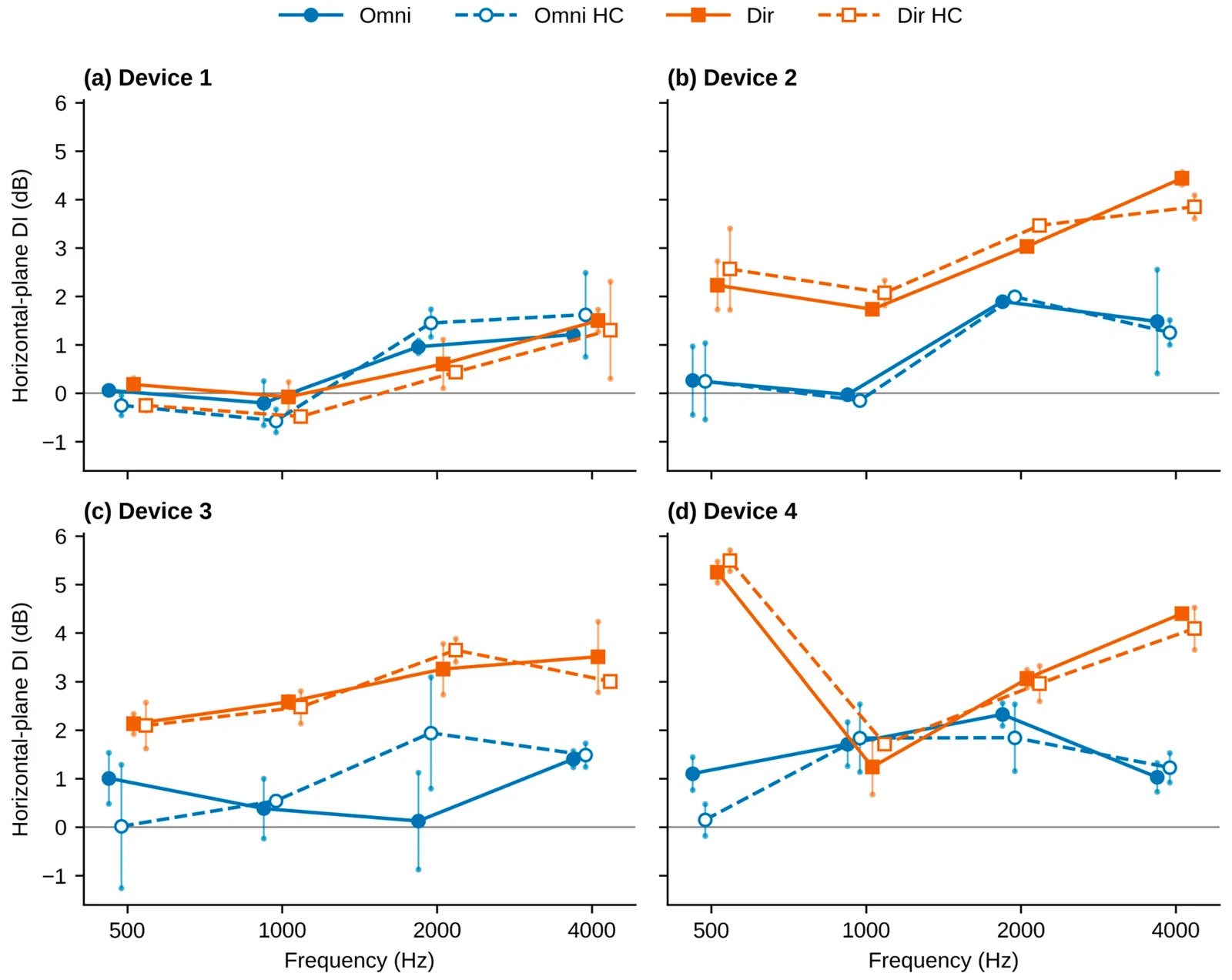
Horizontal-plane directivity index (DI) as a function of test frequency for each hearing aid device under the four combinations of head covering and microphone mode: (**a**) Device 1; (**b**) Device 2; (**c**) Device 3; (**d**) Device 4. Large symbols show the mean of the two technical replicates; small dots joined by thin vertical lines show the individual replicate values. Solid lines indicate uncovered conditions (Omni, Dir) and dashed lines indicate head covering conditions (Omni HC, Dir HC). The horizontal line at 0 dB indicates equal sensitivity at the frontal reference region (30–60°) and averaged across all measured horizontal-plane azimuths; positive values indicate greater relative sensitivity to the frontal reference region, and negative values indicate lower relative sensitivity to that region.

Across the four tested devices, fixed directional mode produced a higher DI than omnidirectional mode on average; however, the magnitude and direction of the difference varied across devices and test frequencies. Mean DI values for every combination of device, frequency, and condition are presented in Table 1 and Figure 3.

### 3.1. Effect of Directionality

Averaged across head covering conditions and test frequencies, mean DI was 0.92 dB in Omni mode and 2.42 dB in Dir mode, corresponding to a mean directional–omnidirectional difference of 1.50 dB. The main effect of microphone mode did not reach statistical significance when the four hearing-aid devices were treated as the independent experimental units, *F* (1,3) = 7.57, *p* = 0.071, partial η^2^ = 0.716. The corresponding mean difference was 1.503 dB (95% CI: −0.236 to 3.242 dB), *t* (3) = 2.75, Cohen’s *d*_z_ = 1.38.

### 3.2. Effect of Head Covering

The overall covered–uncovered change in DI was small, with a mean ΔDI of −0.023 dB (95% CI: −0.193 to 0.148 dB), *t* (3) = −0.43, *p* = 0.699, Cohen’s *d*_z_ = −0.21. The corresponding repeated-measures main effect of head covering was *F* (1,3) = 0.18, *p* = 0.699, partial η^2^ = 0.057. This nonsignificant result indicates that no average covering-related shift was statistically detected across the four devices; it should not be interpreted as evidence of equivalence between covered and uncovered conditions.

The interaction between head covering and frequency was not statistically significant, *F* (3,9) = 1.47, *p* = 0.287, Greenhouse–Geisser adjusted *p* = 0.301, partial η^2^ = 0.329. The interaction between head covering and microphone mode was also not statistically significant, *F* (1,3) = 0.04, *p* = 0.860, partial η^2^ = 0.012.

**Figure 4:**
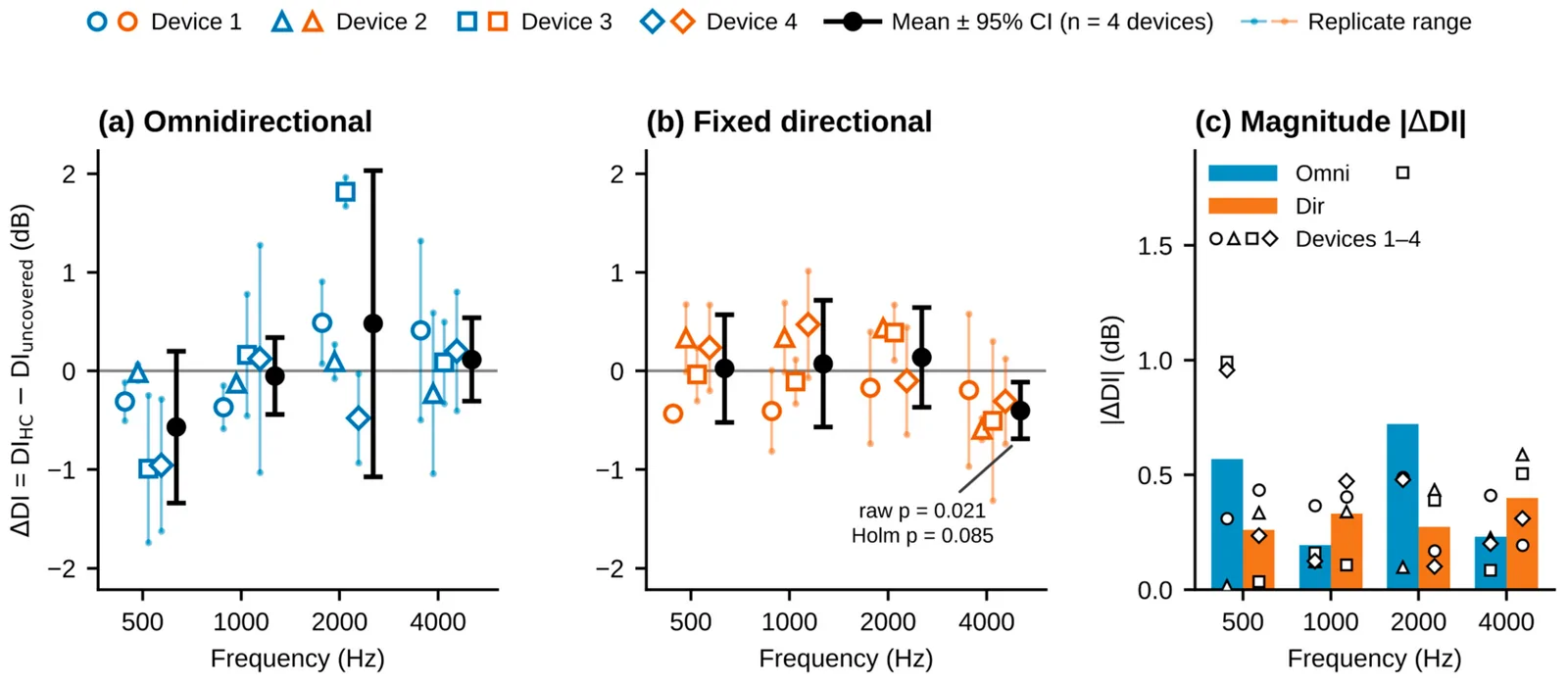
Head covering effect on horizontal-plane DI, expressed as ΔDI = DI (covered) − DI (uncovered), for (**a**) omnidirectional and (**b**) fixed directional modes. Open symbols show device-level ΔDI (mean of two replicates), and thin lines show replicate-level values. Black circles and error bars show the mean ΔDI and 95% CI across devices (*n* = 4). (**c**) Mean |ΔDI| per frequency and mode, with individual devices overlaid. Only the directional 4000 Hz contrast had a CI excluding zero (−0.40 dB, 95% CI −0.69 to −0.11; Holm-adjusted *p* = 0.085).

Planned frequency-specific contrasts showed that no covered–uncovered difference remained statistically significant after correction for multiple comparisons. In Dir mode, mean ΔDI was +0.025 dB at 500 Hz, +0.075 dB at 1000 Hz, +0.138 dB at 2000 Hz, and −0.400 dB at 4000 Hz. The 4000-Hz contrast was significant before correction (*p* = 0.021) but not after Holm correction across the four Dir mode frequency contrasts (*p* = 0.085). The corresponding mean absolute changes were 0.260, 0.331, 0.274, and 0.400 dB, respectively. Device-level and replicate-level ΔDI values with 95% confidence intervals are shown in Figure 4.

### 3.3. Technical Repeatability

**Figure 5:**
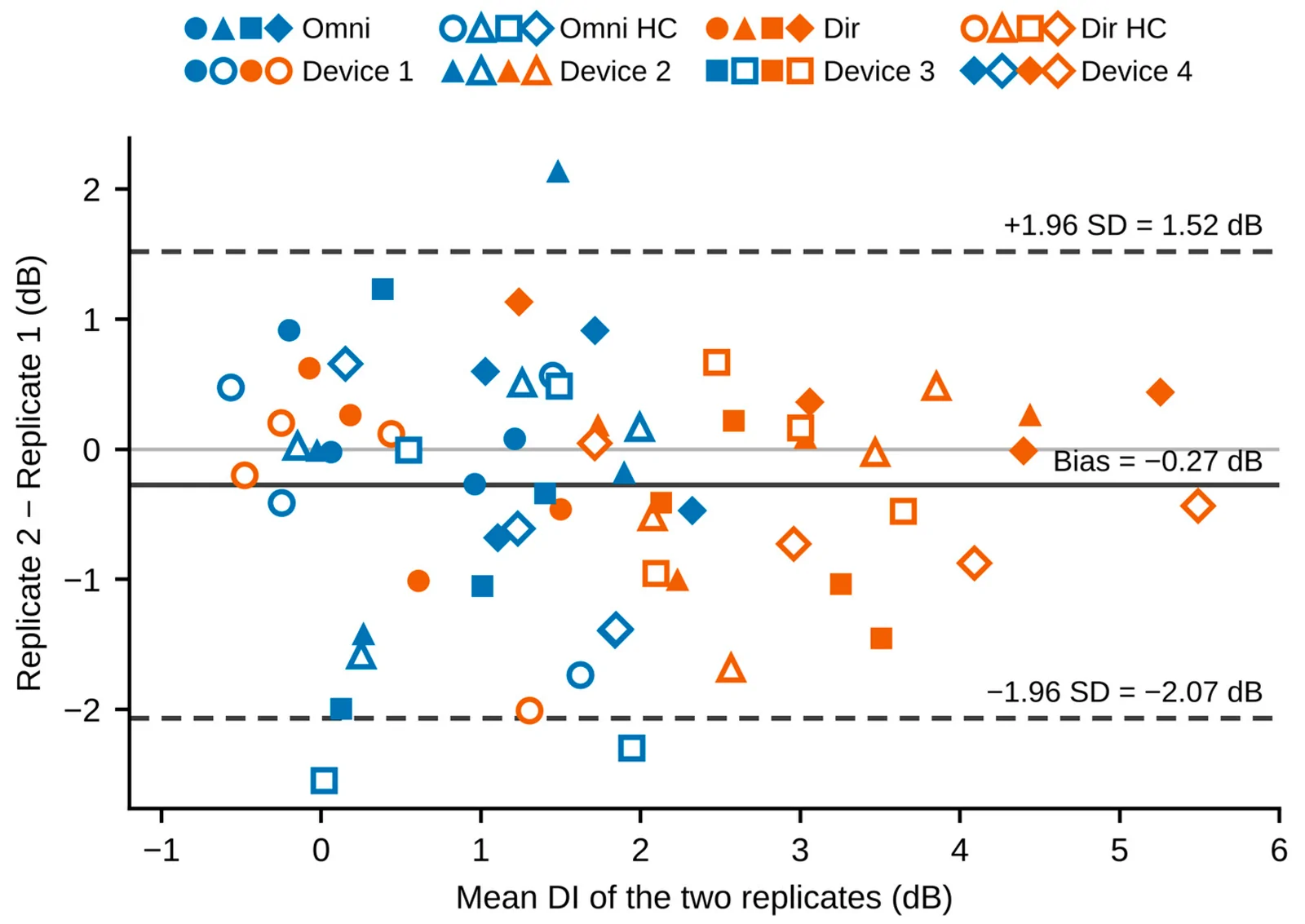
Bland–Altman plot of technical repeatability of horizontal-plane DI across all 64 device × frequency × condition combinations, showing the replicate difference (Replicate 2 − Replicate 1) against the replicate mean. Symbol shape identifies the device. Blue = omnidirectional and orange = fixed directional; open symbols = head covering. Solid line: mean bias (−0.27 dB); dashed lines: 95% limits of agreement (−2.07 to 1.52 dB). Devices and fabric were not repositioned between replicates.

Across all measurement conditions, the mean Replicate 2 − Replicate 1 difference in corrected DI was −0.275 dB. The mean absolute difference between technical repetitions was 0.714 dB, with an SD of replicate differences of 0.915 dB and 95% limits of agreement from −2.07 to 1.52 dB. Because hearing aids and fabric were not repositioned between repetitions, these values reflect acquisition repeatability rather than repositioning variability. Replicate agreement is shown in Figure 5.

### 3.4. Effect of Frequency

Directivity index varied significantly across test frequency, *F* (3, 9) = 8.72, *p* = 0.005, partial η^2^ = 0.744. After Greenhouse–Geisser correction (ε = 0.515), the effect remained statistically significant, *p* = 0.030. Collapsed across microphone mode and head covering condition, mean horizontal-plane DI was 1.39 dB at 500 Hz, 0.92 dB at 1000 Hz, 2.06 dB at 2000 Hz, and 2.30 dB at 4000 Hz. These frequency effects should be interpreted within the specific measurement configuration used in the present study.

### 3.5. Differences Among the Tested Hearing Aid Devices

Directional performance differed across the four tested hearing aid devices. Device 1 showed comparatively small directional responses under several tested conditions. Because the original protocol did not include an independent post hoc microphone function or program state verification, this finding should be interpreted as specific to the tested device and measurement configuration rather than as evidence of generally poorer performance by the corresponding manufacturer.

### 3.6. Interactions

The microphone mode × frequency interaction was not statistically significant, *F* (3,9) = 1.08, *p* = 0.406, partial η^2^ = 0.265. After Greenhouse–Geisser correction, the interaction remained nonsignificant (*p* = 0.381).

The head covering × microphone mode interaction was not statistically significant, *F* (1,3) = 0.04, *p* = 0.860, partial η^2^ = 0.012.

The head covering × frequency interaction was also not statistically significant, *F* (3,9) = 1.47, *p* = 0.287, partial η^2^ = 0.329; after Greenhouse–Geisser correction, *p* = 0.301. The head covering × microphone mode × frequency interaction yielded *F* (3,9) = 4.23, *p* = 0.040 before correction. However, after Greenhouse–Geisser correction, the interaction was not statistically significant (*p* = 0.093, partial η^2^ = 0.585). This interaction was therefore interpreted cautiously as exploratory.

We did not perform inferential interaction tests involving hearing aid device or manufacturer because only one hearing aid device represented each manufacturer.

### 3.7. Polar Patterns

**Figure 6:**
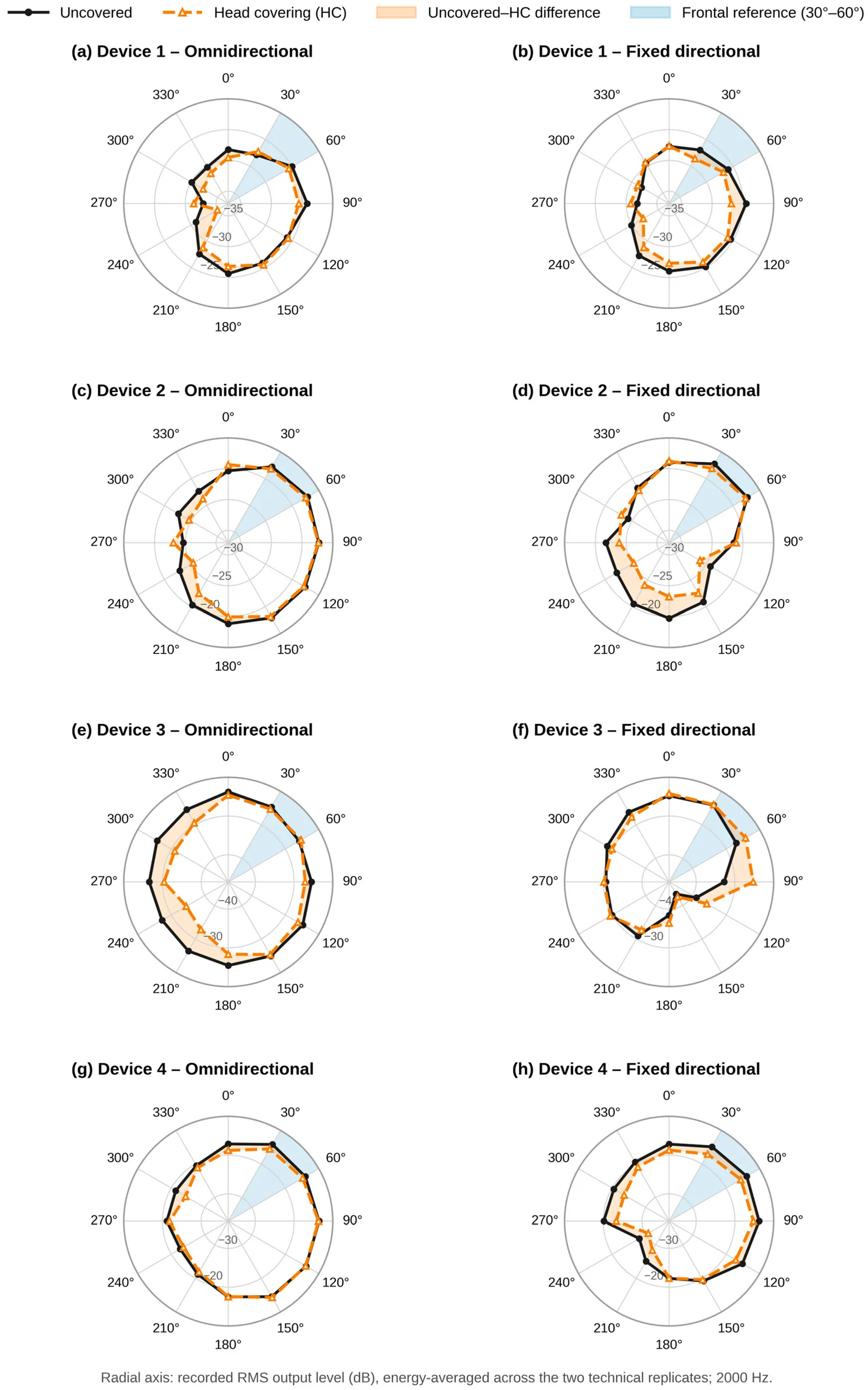
Horizontal-plane polar responses at 2000 Hz for Devices 1–4 (rows) in omnidirectional (**left**) and fixed directional (**right**) modes. Solid black lines show the uncovered condition, dashed orange lines show the head covering (HC) condition, and orange shading marks their difference. The radial axis shows recorded RMS output level (dB), energy averaged across replicates. The blue sector marks the 30–60° frontal reference region; KEMAR was rotated clockwise in 30° steps.

Figure 6 presents the averaged 2000-Hz polar responses for the four tested hearing aid devices, while polar plots at all four test frequencies are provided in Supplementary Figures S1–S4. The polar responses show clear differences between omnidirectional and fixed directional processing for several tested devices. Adding the lightweight cotton covering produced relatively small changes in the overall shape of the directional patterns, although the magnitude and direction of these changes varied across devices. The complete frequency-specific responses show that the covering’s effect was not uniform across frequencies or hearing aid devices. These descriptive patterns were examined quantitatively in the present dataset using the frequency-specific covered–uncovered ΔDI analysis described above.

## 4. Discussion

Fixed directional processing produced a higher DI than omnidirectional processing on average, with a mean difference of 1.50 dB; however, this difference did not reach statistical significance when the four hearing aid devices were treated as the independent experimental units [4,6]. No statistically detectable average difference was observed between the covered and uncovered conditions; however, this finding should not be interpreted as evidence that the two conditions are equivalent. The covered–uncovered change varied across hearing aid devices and frequencies, and the small number of independent devices limits the precision of the overall estimate [5]. Visual inspection of the polar responses suggested small changes in absolute microphone output in some covered conditions; however, these changes were not uniform across devices and were not analyzed as a separate outcome. Whether the observed changes in absolute response are perceptually meaningful requires behavioral investigation.

The absence of a significant head covering effect is consistent with previous work on the impact of hair on directional performance, which likewise showed little or no effect [11]. From an acoustic perspective, the minimal degradation observed here can be attributed to the properties of the thin, porous cotton fabric used, which permits relatively unattenuated transmission of sound across the speech frequency range, particularly at low and mid frequencies [12,16]. Because directional microphones are most beneficial at mid-to-high frequencies (2000–4000 Hz), where spatial cues are strongest [6], it is notable that the head covering had no statistically significant effect on DI at any frequency within this range.

Previous studies of the relationship between directional processing and speech intelligibility in background noise support the finding that directional listening improves SNR and speech intelligibility [4]. Manikin-based DI measurements have also been shown to predict the improvements in speech-in-noise performance that hearing aids achieve in practice [7,20]. The mean directional advantage observed here (1.50 dB), although not statistically significant with four devices, aligns with decades of research showing a positive relationship between directional processing and speech recognition in noise [4,6]. The present findings extend this literature: when adaptive microphone features, head covering, and manufacturer were deliberately controlled, a directional response remained measurable in most tested devices, and the average covering-related change was small, although device-specific changes were observed.

Our finding of no significant head covering effect is consistent with Wagner [11], who reported that hair covering did not significantly impair speech reception thresholds. Although the present study quantified acoustic directivity while Wagner examined perceptual outcomes, both studies suggest that relatively lightweight coverings, whether hair or thin cotton, have limited average effects on the measured directional response, although differences in study design, outcome measures, and tested devices warrant cautious interpretation. The effects of light coverings on sound attenuation and scattering appear to be small on average.

Directional mode DI was highest at 4000 Hz (mean 3.26 dB). The mean Dir–Omni difference was 2.14, 0.97, 0.99, and 1.92 dB at 500, 1000, 2000, and 4000 Hz, respectively; the 500-Hz value was driven mainly by Device 4, and the microphone mode × frequency interaction was not significant (Greenhouse–Geisser *p* = 0.381). The high directional-mode DI at 4000 Hz is consistent with earlier studies reporting that spatial selectivity is greatest in the mid-to-high frequency range [6]. The differences observed among the tested hearing aid devices may reflect differences in microphone port spacing and orientation, device configuration, and implementation of directional processing [6,7,21]. Previous work has demonstrated that directional performance and the relationship between DI and speech-in-noise performance can vary across individual hearing aid models and configurations [21]. Articulation index-weighted DI measures, which weigh DI according to the importance of each frequency for speech understanding, likewise emphasize this region as a predictor of real-world outcomes [6,22]. The differences observed among devices, specifically the lower DI values for Device 1 relative to Devices 2, 3, and 4, may reflect variations in microphone spacing, port placement, housing design, and signal processing [7,21]; however, the present design cannot determine which factor was responsible. Microphone orientation and device placement are known to affect DI values [7]. Furthermore, configurations such as BTE devices with larger microphone spacing and off-ear microphone arrays have produced higher DI values, indicating that physical design plays an important role alongside signal processing [21].

Although the overall effect of the covering was small, the magnitude and direction of ΔDI varied across individual devices and frequencies. In the fixed directional condition at 4000 Hz, ΔDI ranged from approximately −0.20 to −0.59 dB across the four tested devices. At other frequencies, both positive and negative changes were observed. Such device-specific variability could reflect differences in microphone port spacing and orientation, housing geometry, the proximity of the fabric to individual microphone ports, or differences in the implementation of fixed directional processing. Because only one hearing aid device represented each manufacturer, these patterns should be interpreted as device-specific observations rather than manufacturer-wide effects.

The present evaluation used fixed directional programs with all adaptive features deactivated. Therefore, an adaptive device responding to environmental change may not behave the same way under typical real-world conditions [8]. Adaptive devices can improve SNR in environments with varying or moving noise sources by dynamically adjusting array weights to suppress non-stationary interference [6,8] and have been shown to improve measurable aspects of SNR and speech reception thresholds in environments with multiple competing talkers or spatially changing interference [23]. Under real-world listening conditions, adaptive devices may help maintain, or potentially improve, performance when head coverings are worn; targeted studies are needed to establish whether this is the case.

Fixed directional DI values also differed among the four tested devices, with Devices 2, 3, and 4 showing larger values than Device 1 [7,21]. Although the same prescriptive targets were used for every device and all were tested with adaptive processing disabled, compression and other proprietary processing parameters were not matched across devices. These differences may therefore reflect the physical characteristics of the devices (microphone port spacing, port location and housing design), the proprietary algorithms implemented in the fixed directional modes, or other device-specific processing differences, which the present design cannot separate [7,21,24]. Because this study was not designed to compare manufacturers, these observations should not be used to rank manufacturers or their product lines.

### 4.1. Clinical Implications

The present study evaluated directional response rather than absolute microphone sensitivity. Therefore, these data do not support conclusions about changes in overall hearing aid output, perceived loudness, or the need for volume control adjustments when a covering is worn. Future studies should evaluate absolute output or microphone sensitivity as a separate outcome.

The observed differences among the tested devices also demonstrate that directional performance can vary across hearing aid designs. Because only one device configuration represented each manufacturer, these findings should not be interpreted as manufacturer-wide rankings. Device-specific verification may be particularly useful when hearing-in-noise performance is a primary fitting goal.

The present findings characterize the electroacoustic directional response of four specific hearing aid devices under one lightweight cotton configuration. They do not demonstrate preservation of speech intelligibility, speech reception thresholds, output signal-to-noise ratio, or real-world directional benefit. Because the covering-related changes varied across devices and frequencies, device-specific verification may be appropriate when a patient’s typical head covering directly overlies the hearing aid microphone ports.

### 4.2. Limitations

The results are based on a two-layer cotton material and may not generalize to thicker or more tightly woven fabrics. Although the controlled KEMAR setup reduced variability, it does not reflect dynamic real-world listening conditions involving head movement, talker variability, or complex noise. Only fixed directional programs were tested, with adaptive features disabled, and each condition was measured twice, limiting assessment of within-manufacturer device variability. Therefore, differences among devices should not be generalized to broader product portfolios. The study was also not designed to compare manufacturers: compression settings were not matched across devices, and between-device differences may not apply to other models or later product generations.

## 5. Conclusions

Fixed directional processing tended to produce greater horizontal-plane directivity than omnidirectional processing in the hearing aid devices evaluated, although this difference was not statistically significant. The effect of the lightweight cotton covering was generally small on average but varied across hearing aid devices and test frequencies, with larger covered–uncovered changes observed in selected conditions. Because only four hearing aid devices and one fabric configuration were evaluated, and because measurements were obtained in a sound-treated rather than an anechoic environment, the findings should be interpreted as device- and setup-specific electroacoustic observations. The present results do not establish equivalence between covered and uncovered conditions and do not demonstrate preservation of speech-in-noise benefit in real-world listening. Future studies should evaluate multiple devices per model, systematically characterize fabrics and layer configurations, use standards-based acoustic measurements, incorporate adaptive microphone processing, and assess behavioral speech-in-noise outcomes.

## Figures and Tables

**Table 1:** Mean horizontal-plane directivity index (dB) for each tested hearing aid device and frequency under the four experimental conditions: omnidirectional (Omni), omnidirectional with head covering (Omni HC), fixed directional (Dir), and fixed directional with head covering (Dir HC). Values are averaged across the two technical replicates.

Device	Frequency (Hz)	Omni	Omni HC	Dir	Dir HC
1	500	0.06	−0.25	0.18	−0.25
	1000	−0.20	−0.57	−0.08	−0.48
	2000	0.96	1.45	0.61	0.44
	4000	1.21	1.62	1.50	1.30
2	500	0.27	0.25	2.23	2.57
	1000	−0.03	−0.15	1.73	2.07
	2000	1.90	1.99	3.03	3.47
	4000	1.48	1.26	4.44	3.85
3	500	1.01	0.02	2.13	2.10
	1000	0.38	0.55	2.58	2.48
	2000	0.13	1.94	3.26	3.64
	4000	1.40	1.49	3.51	3.00
4	500	1.11	0.15	5.26	5.49
	1000	1.71	1.84	1.24	1.71
	2000	2.32	1.85	3.06	2.96
	4000	1.03	1.23	4.40	4.09

## Data Availability

Replicate-level DI values are provided in Supplementary Table S1. The complete angular recordings are available from the corresponding author upon reasonable request.

## Supplementary Materials

The following supporting information can be downloaded at: https://www.mdpi.com/article/10.3390/audiolres16050152/s1, Figure S1: Polar responses for Device 1 at all test frequencies; Figure S2: Polar responses for Device 2 at all test frequencies; Figure S3: Polar responses for Device 3 at all test frequencies; Figure S4: Polar responses for Device 4 at all test frequencies. Table S1. Replicate-level horizontal-plane directivity index (DI_b_) values for each tested hearing aid device, microphone mode, head covering condition, and frequency.

## Abbreviations

The following abbreviations are used in this manuscript:

BTE	behind-the-ear
DI	directivity index
HC	head covering
KEMAR	Knowles Electronics Manikin for Acoustic Research
NAL-NL2	National Acoustic Laboratories Non-Linear 2 prescriptive formula
RIC	receiver-in-canal
RMS	root-mean-square
SNR	signal-to-noise ratio
SPL	sound pressure level
