# Supplementary material for: Effect of Head Coverings on Directional Microphone Performance in Modern Hearing Aids: A KEMAR-Based Laboratory Evaluation
Source: Audiol Res. 2026 Oct 3;16(5):152. doi: 10.3390/audiolres16050152 (PMC13701770; doi:10.3390/audiolres16050152)
Supplement: Supplementary file 1 [file audiolres-16-00152-s001.zip › audiolres-4561586-supplementary.pdf]

Supplementary Materials

Effect of Head Coverings on Directional Microphone Performance in Modern Hearing Aids: A KEMAR-Based Laboratory Evaluation

Contents

Figure S1. Polar responses for Device 1 at all test frequencies.

Figure S2. Polar responses for Device 2 at all test frequencies.

Figure S3. Polar responses for Device 3 at all test frequencies.

Figure S4. Polar responses for Device 4 at all test frequencies.

Table S1. Replicate-level horizontal-plane directivity index (DI_h) values for each tested hearing aid device, microphone mode, head covering condition, and frequency.

Angles in all figures are given in the azimuth coordinate system used during data collection, with KEMAR rotated clockwise in 30° steps. Device numbering matches the main manuscript.

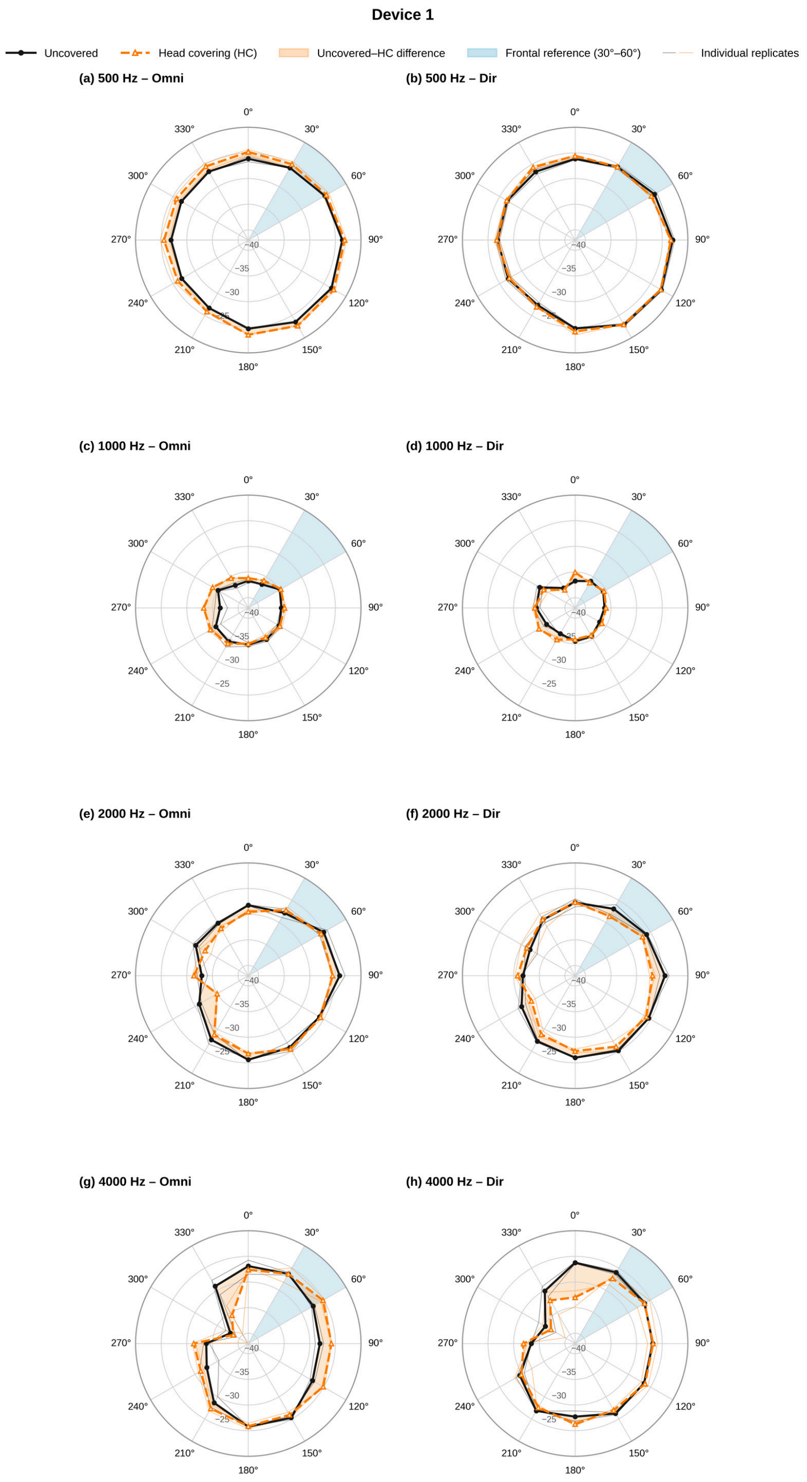

Figure S1. *Horizontal-plane polar responses for Device 1 at 500, 1000, 2000, and 4000 Hz (rows) in omnidirectional (left) and fixed directional (right) modes. Thick lines show the replicate-averaged response: solid black for uncovered and dashed orange for head covering (HC). Thin lines show individual replicates, and orange shading marks the uncovered–HC difference. The blue sector marks the 30°–60° frontal reference region, and the radial axis shows recorded RMS output level (dB) on a common scale across panels.*

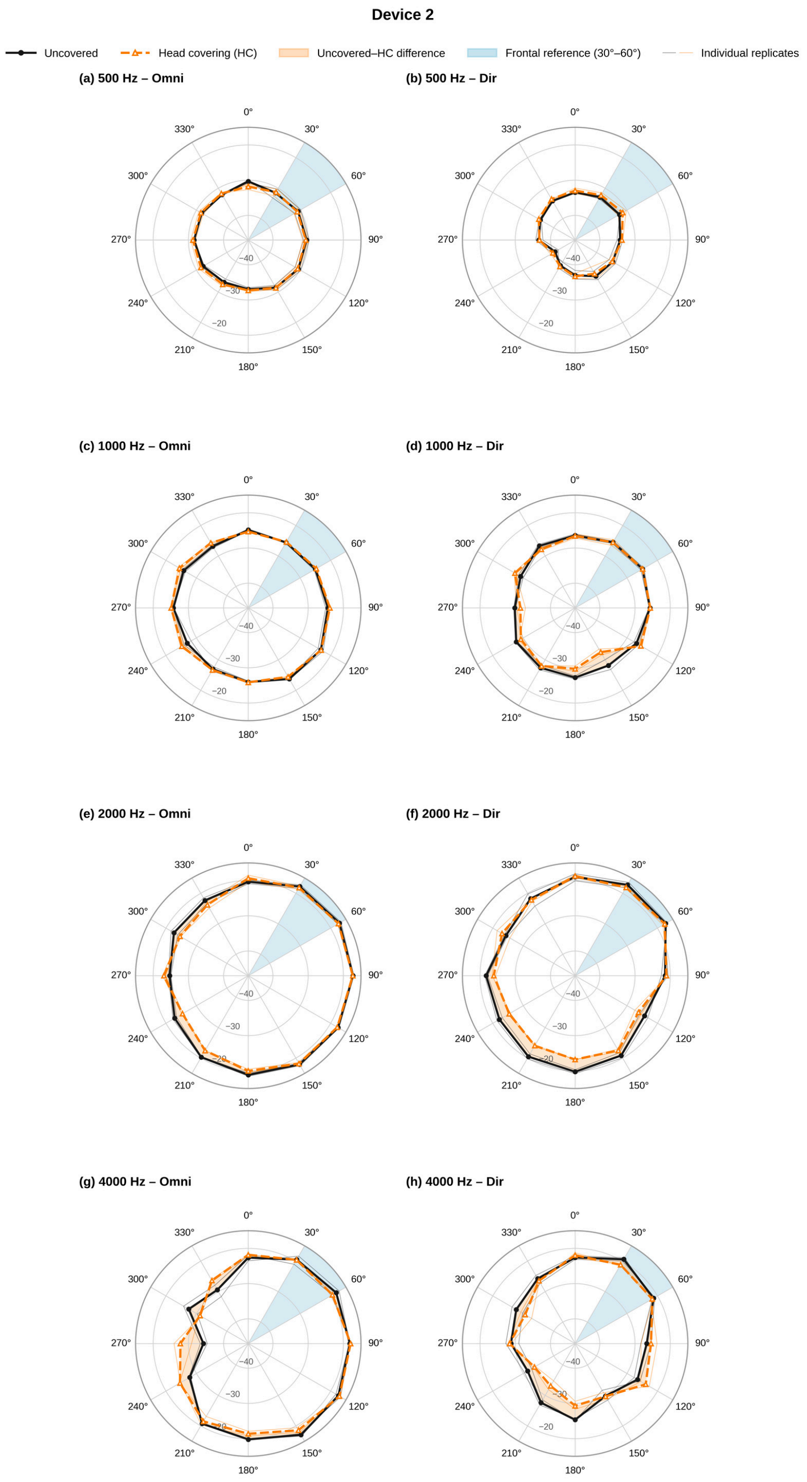

Figure S2. *Horizontal-plane polar responses for Device 2 at all four test frequencies. Conventions as in Figure S1.*

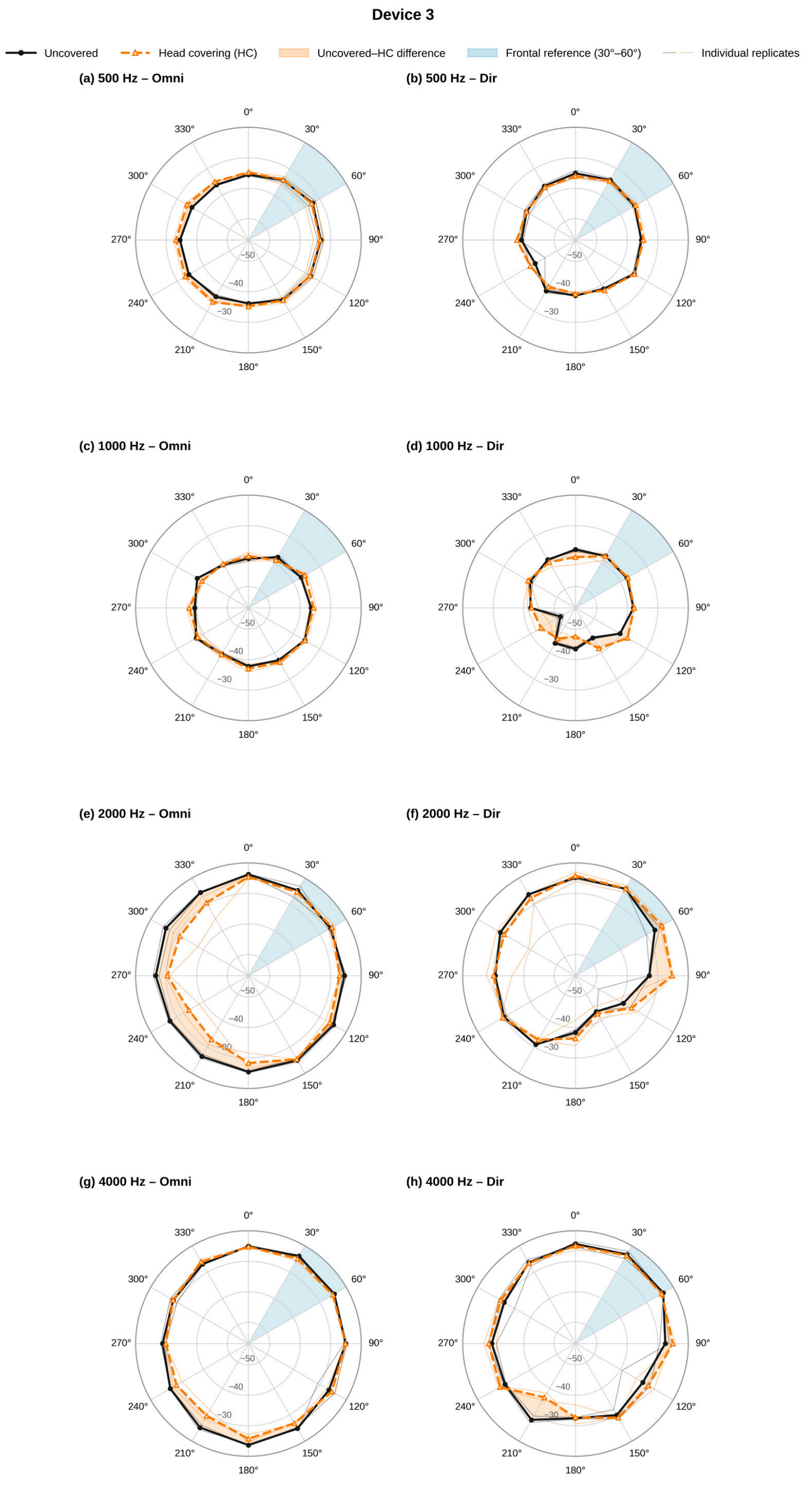

Figure S3. *Horizontal-plane polar responses for Device 3 at all four test frequencies. Conventions as in Figure S1.*

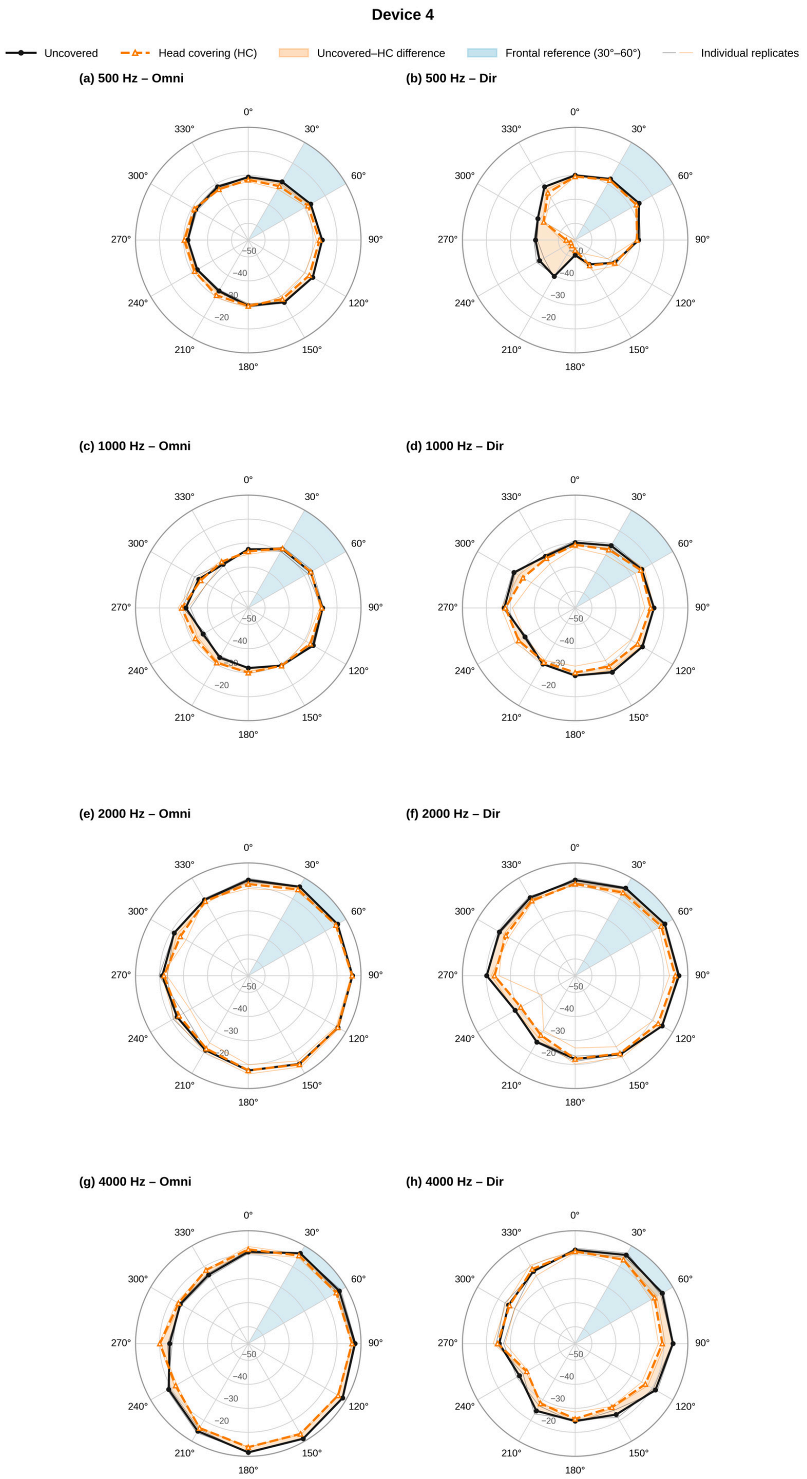

Figure S4. *Horizontal-plane polar responses for Device 4 at all four test frequencies. Conventions as in Figure S1.*

Supplementary Table S1

Replicate-level horizontal-plane directivity index (DI_h) values for each tested hearing aid device, microphone mode, head covering condition, and frequency.

Replicates 1 and 2 are technical repetitions; the hearing aids and fabric were not repositioned between repetitions.

Device	Frequency (Hz)	Microphone mode	Head-covering condition	Replicate 1 DI_h (dB)	Replicate 2 DI_h (dB)	Mean DI_h (dB)
Device 1	500	Omnidirectional	Uncovered	0.071	0.051	0.061
Device 1	500	Omnidirectional	Covered	−0.044	−0.453	−0.249
Device 1	500	Fixed directional	Uncovered	0.049	0.313	0.181
Device 1	500	Fixed directional	Covered	−0.355	−0.150	−0.253
Device 1	1000	Omnidirectional	Uncovered	−0.660	0.258	−0.201
Device 1	1000	Omnidirectional	Covered	−0.805	−0.330	−0.567
Device 1	1000	Fixed directional	Uncovered	−0.387	0.236	−0.075
Device 1	1000	Fixed directional	Covered	−0.380	−0.579	−0.479
Device 1	2000	Omnidirectional	Uncovered	1.094	0.828	0.961
Device 1	2000	Omnidirectional	Covered	1.167	1.734	1.450
Device 1	2000	Fixed directional	Uncovered	1.112	0.102	0.607
Device 1	2000	Fixed directional	Covered	0.377	0.498	0.437
Device 1	4000	Omnidirectional	Uncovered	1.171	1.252	1.212
Device 1	4000	Omnidirectional	Covered	2.490	0.756	1.623
Device 1	4000	Fixed directional	Uncovered	1.729	1.270	1.500
Device 1	4000	Fixed directional	Covered	2.308	0.302	1.305
Device 2	500	Omnidirectional	Uncovered	0.974	−0.442	0.266
Device 2	500	Omnidirectional	Covered	1.039	−0.540	0.250
Device 2	500	Fixed directional	Uncovered	2.730	1.732	2.231
Device 2	500	Fixed directional	Covered	3.406	1.726	2.566
Device 2	1000	Omnidirectional	Uncovered	−0.024	−0.028	−0.026
Device 2	1000	Omnidirectional	Covered	−0.162	−0.136	−0.149
Device 2	1000	Fixed directional	Uncovered	1.639	1.828	1.733
Device 2	1000	Fixed directional	Covered	2.330	1.815	2.073
Device 2	2000	Omnidirectional	Uncovered	1.984	1.808	1.896
Device 2	2000	Omnidirectional	Covered	1.908	2.078	1.993
Device 2	2000	Fixed directional	Uncovered	2.986	3.079	3.032
Device 2	2000	Fixed directional	Covered	3.480	3.454	3.467
Device 2	4000	Omnidirectional	Uncovered	0.412	2.553	1.482
Device 2	4000	Omnidirectional	Covered	1.000	1.512	1.256
Device 2	4000	Fixed directional	Uncovered	4.306	4.572	4.439
Device 2	4000	Fixed directional	Covered	3.608	4.092	3.850
Device 3	500	Omnidirectional	Uncovered	1.536	0.484	1.010
Device 3	500	Omnidirectional	Covered	1.290	−1.255	0.018
Device 3	500	Fixed directional	Uncovered	2.337	1.924	2.130
Device 3	500	Fixed directional	Covered	2.571	1.620	2.095
Device 3	1000	Omnidirectional	Uncovered	−0.232	0.999	0.384

Device	Frequency (Hz)	Microphone mode	Head-covering condition	Replicate 1 DI_h (dB)	Replicate 2 DI_h (dB)	Mean DI_h (dB)
Device 3	1000	Omnidirectional	Covered	0.548	0.542	0.545
Device 3	1000	Fixed directional	Uncovered	2.474	2.693	2.584
Device 3	1000	Fixed directional	Covered	2.141	2.809	2.475
Device 3	2000	Omnidirectional	Uncovered	1.125	−0.872	0.126
Device 3	2000	Omnidirectional	Covered	3.090	0.796	1.943
Device 3	2000	Fixed directional	Uncovered	3.774	2.736	3.255
Device 3	2000	Fixed directional	Covered	3.882	3.406	3.644
Device 3	4000	Omnidirectional	Uncovered	1.574	1.234	1.404
Device 3	4000	Omnidirectional	Covered	1.243	1.732	1.488
Device 3	4000	Fixed directional	Uncovered	4.235	2.782	3.508
Device 3	4000	Fixed directional	Covered	2.920	3.084	3.002
Device 4	500	Omnidirectional	Uncovered	1.446	0.765	1.106
Device 4	500	Omnidirectional	Covered	−0.179	0.478	0.150
Device 4	500	Fixed directional	Uncovered	5.036	5.474	5.255
Device 4	500	Fixed directional	Covered	5.707	5.274	5.491
Device 4	1000	Omnidirectional	Uncovered	1.257	2.170	1.714
Device 4	1000	Omnidirectional	Covered	2.534	1.140	1.837
Device 4	1000	Fixed directional	Uncovered	0.674	1.805	1.239
Device 4	1000	Fixed directional	Covered	1.689	1.735	1.712
Device 4	2000	Omnidirectional	Uncovered	2.561	2.088	2.325
Device 4	2000	Omnidirectional	Covered	2.535	1.155	1.845
Device 4	2000	Fixed directional	Uncovered	2.879	3.241	3.060
Device 4	2000	Fixed directional	Covered	3.322	2.595	2.959
Device 4	4000	Omnidirectional	Uncovered	0.729	1.328	1.029
Device 4	4000	Omnidirectional	Covered	1.533	0.924	1.229
Device 4	4000	Fixed directional	Uncovered	4.405	4.393	4.399
Device 4	4000	Fixed directional	Covered	4.527	3.652	4.089
